# Transcriptional Inhibition of *Sp-IAG* by Crustacean Female Sex Hormone in the Mud Crab, *Scylla paramamosain*

**DOI:** 10.3390/ijms21155300

**Published:** 2020-07-26

**Authors:** Qingling Jiang, Bei Lu, Guizhong Wang, Haihui Ye

**Affiliations:** College of Ocean and Earth Sciences, Xiamen University, Xiamen 361102, China; jangqingling@163.com (Q.J.); beilu@stu.xmu.edu.cn (B.L.); gzwang@xmu.edu.cn (G.W.)

**Keywords:** insulin-like androgenic hormone, transcription regulation, crustacean female sex hormone, crustacean

## Abstract

In crustaceans, the regulation of sex differentiation is mediated by insulin-like androgenic hormone (IAG) and crustacean female sex hormone (CFSH). CFSH is reported to inhibit IAG gene (*Sp-IAG*) expression in the mud crab *Scylla paramamosain*, but the regulatory mechanism is not well understood. A 2674 bp 5′ flanking *Sp-IAG* contains many potential transcription factor binding sites. In this study, analysis of serially deleted 5′ flanking *Sp-IAG* and site-directed mutation (SDM) of transcription factor binding sites of the same gene showed that the promoter activity of reporter vectors with Sox-5-binding site, signal transducers and activators of transcription (STAT)-binding site and activator protein 1 (AP-1)-binding site were significantly higher than that of vectors without these regions, suggesting that they were involved in transcriptional regulation of *Sp-IAG* expression. The expression analysis of these transcription factor showed that there was no difference in the level of mRNA in *Sox-5* and *AP-1* in androgenic gland treated with recombinant CFSH, but expression of *Sp-STAT* was significantly reduced, suggesting that CFSH regulates the expression of *Sp-STAT*, inhibiting its function to regulate *Sp-IAG*. Further experiment revealed that RNAi mediated *Sp-STAT* gene knockdown reduced the expression of *Sp-IAG*. These results suggested that *Sp-*CFSH regulates *Sp-IAG* by inhibiting STAT. This is a pioneering finding on the transcriptional mechanism of *IAG* gene in crustaceans.

## 1. Introduction

Sexually dimorphic traits are widespread in various species, and rely on changes initiated and maintained by key reproductive hormones [1,2]. In male crustaceans only, sex differentiation is largely controlled by the androgenic gland (AG) [3]. The role of AG was first reported in amphipod *Orchestia gammarellus* [4]. Later, cDNA for androgenic gland hormone (AGH) from terrestrial isopod *Armadillidium vulgare* was isolated and characterized [5]. Removing AG or silencing the gene for AGH, as well as AG implantation or injection of AG extracts has been shown to affect primary and secondary sex characteristics in various crustacean species, including *A. vulgare*, the red-claw crayfish *Cherax quadricarinatus* and the giant freshwater prawn *Macrobrachium rosenbergii* [6,7,8,9,10,11]. For instance, in *M. rosenbergii*, removal of the AG caused sex reversal in juvenile males, while silencing *Mr-AGH* resulted in hypertrophy of AG and arrested testicular spermatogenesis [12,13].

In the mud crab *Scylla paramamosain*, AGH, also termed AG-specific insulin-like peptide (IAG), is widely distributed in various tissues of both sexes, and may be involved in ovarian development, somatic growth and mating behavior [14,15]. X-organ-sinus gland in eyestalk negatively regulates expression of *Sp-IAG* [15,16]. For the first time, Zhang et al. cloned 5′- region flanking *Sp-IAG*, and found many potential transcription factor binding sites, such as Sox-5, GATA-1, GATA-2, activator protein 1 (AP-1), and signal transducers and activators of transcription (STAT) [15]. Sox is a series of conserved genes, found in many species such as mammals, fish, reptiles, amphibians, birds, and insects, in which Sox-3, Sox-5, Sox-9, and Sox-17 regulate determination of male sex [17,18,19]. GATA proteins are important transcriptional regulators and play an important role in steroidogenesis, sex determination and differentiation [20,21,22,23].

Crustacean female sex hormone (CFSH) was first isolated from eyestalk of the Atlantic blue crab *Callinectes sapidus*, and is integral in the development of female secondary sexual characteristics including parental care and mating system [24]. In *S. paramamosain*, *Sp-CFSH* positively regulates formation of gonopores in females and inhibits expression of *Sp-IAG* in males [2,16]. It is interesting that CFSHs are homologous to interleukin-17 (IL-17) and highly conserved among brachyuran crabs [16]. Although structural homology does not always represent functional similarity, studies on the IL-17 signaling pathway could provide insight on how CFSH mediates its regulation function. Previous studies showed that IL-17 could influence transcription regulators such as AP-1, STAT, NF-κB (nuclear factor kappa-B), thereby controlling expression of specific downstream genes in mammals [25,26,27,28].

IAG and CFSH jointly regulate sex differentiation, and understanding their interaction can provide insight on how they perform their function in crustaceans. To date, there are no reports on the mechanism of IAG inhibition by CFSH. Here, this process was explored in *S. paramamosain* by analyzing transcriptional mechanism of IAG and expression of transcription factors in AGs treated with CFSH in vitro.

## 2. Results

### 2.1. Sp-IAG Promoter Activity

**Figure 1:**
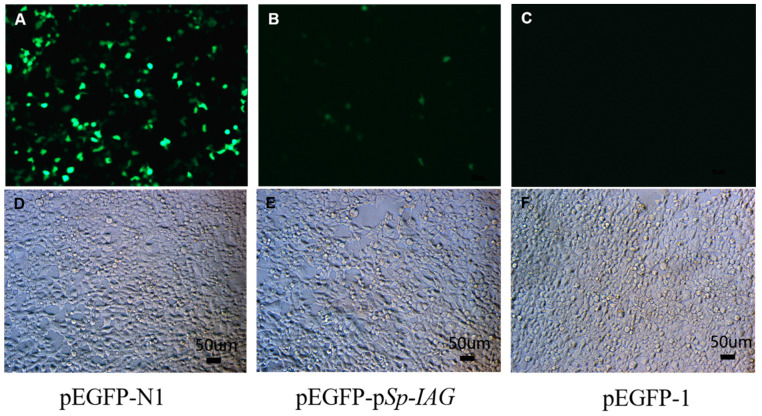
The expression of green fluorescence (EGFP) in human embryonic kidney cell line HEK293FT cells. EGFP were detected in HEK293FT cells transfected with pEGFP-N1 (**A**), pEGFP-p*Sp-IAG* (**B**), and pEGFP-1 (**C**). pEGFP-N1 and pEGFP-1 were used as positive control and negative control, respectively. After transfected for 24 h, the green fluorescence (EGFP) can be detected in (**A**) and (**B**) (positive control and target plasmid), but not in (**C**) (negative control) under a fluorescence microscope. Bright fields are documented in (**D**), (**E**), and (**F**) separately. **A** and **D**, **B** and **E**, **C** and **F** are with the same scale bar, respectively.

The green fluorescence (EGFP) were detected in human embryonic kidney cell line HEK293FT cells after transfection for 24 h, the results showed that EGFP can be detected in cells transfected with pEGFP-N1 (Figure 1A) and pEGFP-p*Sp-IAG* (Figure 1B), but not in cells transfected with pEGFP-1 (Figure 1C). pEGFP-N1 used as a positive control contains cytomegalovirus (CMV) promoter, which can effectively turn on the expression of EGFP. EGFP in cells transfected with pEGFP-p*Sp-IAG* demonstrated promoter activity at 5′-flanking sequence, while pEGFP-1 serving as negative control did not produce any fluorescence. Cells in bright fields are observed and shown in Figure 1D–F, separately.

### 2.2. Analysis of Promoter Activity by Serial Deletion of 5′-Flanking Region

**Figure 2:**
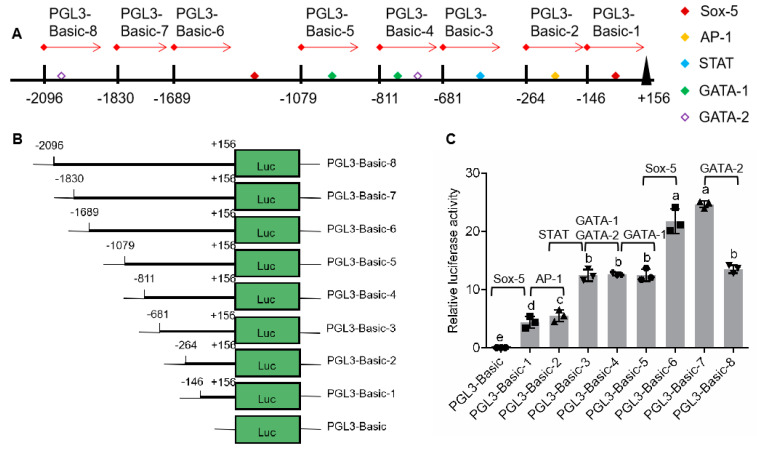
Analysis of promoter activity by serial deletion of 5′-flanking region. (**A**) Schematic diagram on transcription regulatory binding sites. (**B**) Schematic diagram on serial deletion of *Sp-IAG* 5′-flanking region. (**C**) Analysis of relative luciferase activity; pGL3-basic was the negative control. The data are presented as mean ± standard error of mean (SEM) (*n* = 3) with different letters indicating significant differences at *P* < 0.05.

According to distribution of binding sites for transcription factor in 5′-flanking sequence (Figure 2A), eight plasmids, pGL3-p*Sp-IAG*1 to pGL3-p*Sp-IAG*8, were constructed using pGL3-Basic and various regions of 5′-flanking sequence obtained by fixing the 3′ end and extending the 5′ end (Figure 2B). The sequencing results showed that the regions had no mutation and their lengths were 302, 420, 837, 967, 1235, 1845, 1986, and 2252 bp, respectively. Plasmids were transfected on HEK293FT cells, and luciferase activities of Firefly and Renilla determined to analyze the promoter activity. It was shown that the promoter activities of the eight plasmids were significantly higher than that of negative control (pGL3-basic) (Figure 2C). Compared with the previous plasmid, promoter activities were significantly enhanced in pGL3-p*Sp-IAG*1 (Sox-5-binding site), pGL3-p*Sp-IAG*2 (AP-1-binding site), pGL3-p*Sp-IAG*3 (STAT-binding site), and pGL3-p*Sp-IAG*6 (Sox-5-binding site), while there were no significant differences in pGL3-p*Sp-IAG*4 (GATA-1-binding site, GATA-2-binding site), pGL3-p*Sp-IAG*5 (GATA-1-binding site), and pGL3-p*Sp-IAG*7. The promoter activity of pGL3-p*Sp-IAG*8 (GATA-2-binding site) was significantly reduced than that of pGL3-p*Sp-IAG*7.

### 2.3. Analysis of Promoter Activity by Site-Directed Mutation (SDM)

**Figure 3:**
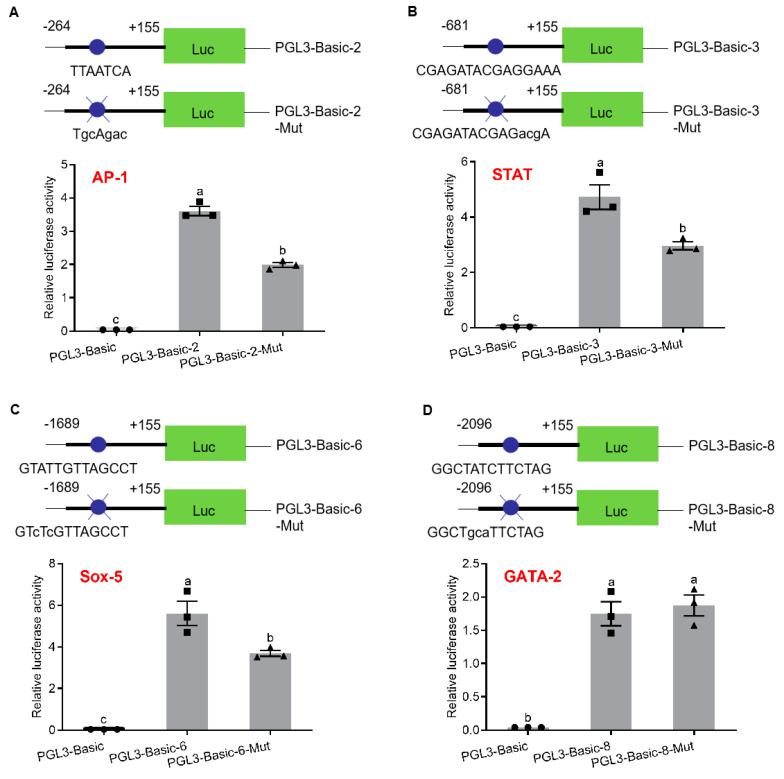
Analysis of binding site of transcription factors with site-directed mutation (SDM). Schematic diagram of reporter vectors and analysis of relative luciferase activity of activator protein 1 (AP-1)-binding site (**A**), signal transducers and activators of transcription (STAT)-binding site (**B**), Sox-5-binding site (**C**) and GATA-2-binding site (**D**). pGL3-Basic was the negative control. Data are presented as mean ± SEM (*n* = 3) with different letters indicating significant differences at *p* < 0.05. The blue dots mark transcription factor binding sites, and blue dots and blue X represent transcription factor binding sites with SDM.

To analyze the regulation of transcription factor binding sites on promoter activity, SDM were performed on AP-1-binding site (pGL3-p*Sp-IAG*2), STAT-binding site (pGL3-p*Sp-IAG*3), Sox-5-binding site (pGL3-p*Sp-IAG*6) and GATA-2-binding site (pGL3-p*Sp-IAG*8). According to the sequencing results, plasmids with SDM were selected to detect the promoter activity. The results showed that promoter activities were significantly reduced in pGL3-p*Sp-IAG*2-Mut (AP-1-binding site: TGCAGAC) (Figure 3A), pGL3-p*Sp-IAG*3-Mut (STAT-binding site: CGAGATACGAGACGA) (Figure 3B), and pGL3-p*Sp-IAG*6-Mut (Sox-5-binding site: GTCTCGTTAGCCT) (Figure 3C), while there was no significant difference in pGL3-p*Sp-IAG*8-Mut (GATA-2-binding site: GGCTGCATTCTAG) (Figure 3D).

### 2.4. Analysis of Gene Expression in AG Treated by rCFSH In Vitro

**Figure 4:**
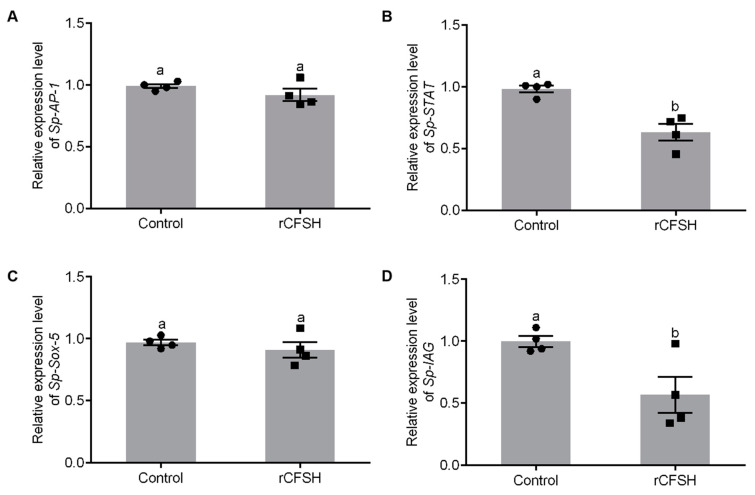
Analysis of transcription factors expression in the androgenic gland (AG). The expression of AP-1 (**A**), STAT (**B**), Sox-5 (**C**), and *Sp-IAG* (**D**). Control: The AGs of control group; rCFSH: The AGs treated with 10^−6^ M rCFSH. The data are presented as mean ± SEM (*n* = 4) with different letters indicating statistical significance at *p* < 0.05.

The precipitate was resuspended in binding buffer with 8 M urea, then analyzed by 15% SDS-PAGE (Figure S1A) which showed that rCFSH (~20 kDa) was expressed in *E. coli* BL21. Figure S1B showed that the purified rCFSH was analyzed by 15% SDS-PAGE, while Figure S1C showed that the renatured protein was analyzed by Western blotting with prepared anti-His-tag rabbit antibody, which confirmed that the antibody could specifically bind to the purified protein. Based on analysis of promoter activity by SDM, AP-1-binding site, STAT-binding site, and Sox-5-binding site may be involved in the regulation of *Sp-IAG* expression. Therefore, AGs were cultured in vitro and treated with rCFSH to further analyze transcriptional regulation of *Sp-IAG* expression by rCFSH. qRT-PCR findings showed that expression of *Sp-STAT* (Figure 4B) and *Sp-IAG* (Figure 4D) were significantly reduced in AG treated with rCFSH, while there was no difference in the expression of *Sp-AP-1* (Figure 4A) and *Sp-Sox* (Figure 4C).

### 2.5. Knockdown of Sp-STAT Reducing Expression of Sp-IAG

**Figure 5:**
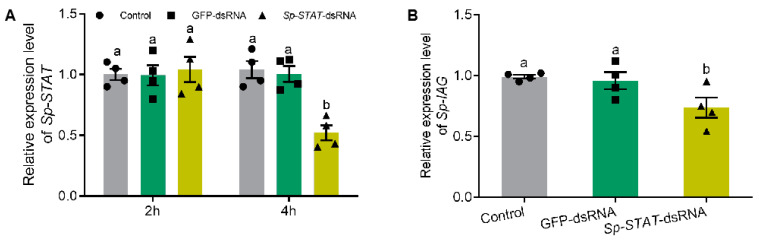
Effect of *Sp-STAT* dsRNA on *Sp-IAG* expression in vitro. Expression of *Sp-STAT* analyzed at hour 2 and 4 (**A**). The expression of *Sp-IAG* was analyzed after incubated for 4 h, water and *GFP* (green fluorescent protein gene) were used as blank and negative controls, respectively (**B**). Data presented as mean ± SEM (*n* = 4). Different letters indicate statistical significance at *p* < 0.05.

The role of *Sp-STAT* was examined by studying the loss of function of this gene by RNAi (dsRNA). Silencing outcomes showed that *Sp-STAT-dsRNA* administration significantly reduced the expression of *Sp-STAT* at 4 h, which was approximately 55% of those treated with saline or *GFP* (green fluorescent protein gene)*-dsRNA* (Figure 5A). qRT-PCR performed to analyze expression of *Sp-IAG* showed that the expression of *Sp-IAG* was also significantly reduced in AG treated with *Sp-STAT-dsRNA* for 4h (Figure 5B).

## 3. Discussion

IAG regulates various reproductive processes such as spermatogenesis, sex differentiation, and sexual shifts in crustaceans [14]. However, the transcriptional regulation of *Sp-IAG* expression is poorly understood. Previous studies established the endocrine axis of the eyestalk-AG-testis in male crustaceans [29,30]. AG is regulated by negative control of the eyestalk, however, the exact mechanism(s) for this inhibition is not well understood [29]. *Sp-CFSH* exclusively expressed in eyestalk ganglion can inhibit expression of *Sp-IAG* in *S. paramamosain*. Therefore, it is important to understand this mutual regulation to shed more insights on the endocrine axis of the eyestalk-AG-testis.

In this study, the HEK293FT cells were transfected with pEGFP-p*Sp-IAG* to determine the promoter activity of the 5′-flanking sequence, and the promoter activity was demonstrated by green fluorescence protein (GFP) expression. Previous studies have shown that the promoter activity of genes in *S. paramamosain* can be analyzed in HEK293FT cells [31,32]. The Figure 1 shows the expression of GFP in HEK293FT cells. However, this expression is rather low for pEGFP-p*Sp-IAG* (Figure 1B), suggesting that the lower transfection efficiency or weaker promoter activity of pEGFP-p*Sp-IAG*. Usually, bigger plasmid bring about lower transfection efficiency. Therefore, compared with pEGFP-N1 (4733 bp), the transfection efficiency of pEGFP-p*Sp-IAG* (6816 bp) could be greatly reduced. In addition, promoter activity is another factor affecting protein expression. Weak promoter activity leads to low GFP expression, while low fluorescence that does not meet detection criteria may be another reason for the small number of green HEK293FT cells transfected with pEGFP-p*Sp-IAG*. Combined with potential transcriptional factor binding sites in 5′ end, GATA was predicted to be involved in transcription regulation of *Sp-IAG*. In this study, the promoter activity of regions flanking 5′ *Sp-IAG* end were analyzed by serial deletion, and it was shown that there was no difference in the promoter activity in pGL3-p*Sp-IAG*5 (GATA-1-binding site) and pGL3-p*Sp-IAG*4 plasmids, suggesting that GATA-1-binding site in p*Sp-IAG*5 may not participate in regulating promoter activity. The promoter activity of pGL3-p*Sp-IAG8* was significantly stronger reduced than that of pGL3-p*Sp-IAG7*, indicating that GATA-2-binding site may have been a negative regulatory element in pGL3-p*Sp-IAG8*. However, analysis of SDM showed that the promoter activity of pGL3-p*Sp-IAG*8-Mut was not significantly different from that of pGL3-p*Sp-IAG*8, suggesting that the decrease in promoter activity of pGL3-p*Sp-IAG8* was not regulated by GATA-2-binding site in p*Sp-IAG*8.

In this study, promoter activity was significantly increased in pGL3-p*Sp-IAG*1 (Sox-5-binding site) and pGL3-p*Sp-IAG*6 (Sox-5-binding site) than in previous plasmids (pGL3-basic and pGL3-p*Sp-IAG*5), suggesting that Sox-5-binding site may participate in enhancing this activity, consistent with SDM results of decreased promoter activity in pGL3-p*Sp-IAG*6-Mut. Sox is an important factor for male sex determination/differentiation in vertebrates or crustaceans [33,34]. In zebra fish, Sox-5 can bind the promoter for *Dmrt1* (doublesex and mab-3-related transcription factor 1) inhibiting expression of the gene, which may influence the differentiation of gender [35]. In medaka, Sox-5 regulates expression of *dmrt1bY*. Moreover, knockout of Sox-5 reverses female to male [36]. Presence of Sox-5-binding site in the 5′-flanking region could explain the association of IAG and male sex differentiation, spermatogenesis and reproductive strategy in *S. paramamosain*. However, expression of *Sp-Sox* was not significantly difference in AG treated by rCFSH, suggesting that Sox-5 does not influence CFSH-mediated transcriptional regulation of *Sp-IAG*. To date, 5′-flanking regions for IAG have only been documented in the Chinese shrimp *Fenneropenaeus chinensis* and the oriental river prawn *Macrobrachium nipponense* and both contain cis-acting element of Sox-5-binding site, suggesting that in decapods, the transcriptional regulation mechanism of IAG may be conserved [15,37].

CFSHs are homologous to interleukin-17 (IL-17) and are highly conserved among brachyuran crabs [16]. Although structural homology does not necessarily represent functional similarity, studies on IL-17 signaling pathway can provide insight on CFSH signaling pathway. Previous studies showed that IL-17, as pro-inflammatory cytokine, is involved in innate and adaptive immunity in vertebrates [38,39]. It has been found that IL-17 regulates genes expression by inducing transcription regulators such as AP-1, STAT, and NF-κB [25,26,28,40,41]. Combined with the results on potential transcriptional factor binding sites in 5′ flanking region of *Sp-IAG*, AP-1-binding site and STAT-binding site were analyzed to explore transcriptional regulation of *Sp-*CFSH on *Sp-IAG* expression.

It was found that AP-1-binding site was involved in the regulation of *Sp-IAG* expression by activating regulation of promoter activity in regions flanking 5′ end of the gene. Serial deletion showed that promoter activity was significantly increased in pGL3-p*Sp-IAG*2 (AP-1-binding site) than (pGL3-p*Sp-IAG*1) plasmid, suggesting that AP-1-binding site may participate in enhancing the promoter activity, consistent with SDM findings on AP-1-binding site. In vertebrates, AP-1 is regarded as an intracellular transcriptional activation factor, regulating expression of target genes, in response to various stimulants on cells, such as growth factors, cytokines, bacterial and viral infections, but also regulates many cellular processes, including differentiation, cell proliferation, inflammation, and other cellular functions [42,43,44]. Studies show that JNK, ERKs, p38MAPK and PI3K/AKT signal transduction pathway regulates activity of AP-1 at both gene and protein level through phosphorylation of different substrates in rodent [45]. In this study, the level of *Sp-AP-1* mRNA was not significantly different in AG treated with rCFSH, suggesting that expression of *Sp-AP-1* at gene level is not involved in CFSH-mediated transcriptional regulation of *Sp-IAG* expression.

Janus kinase (JAK) signal transducer and activator of transcription (STAT) pathway is one of the main signaling pathways in eukaryotic cells, which controls many biological processes. In *Drosophila*, this pathway controls segmentation, eye development, immune response, sex determination, germ/stem cell development, and heterochromatin stability [46,47,48]. JAK/STAT pathway controls expression of insulin-like peptide 8 (*dilp8*) in *Drosophila* to coordinate regenerative disc growth with organisms’ developmental timing [49]. Additionally, JAK/STAT signaling is required in male-germline stem-cell maintenance, germ-cell function and migration in *Drosophila* [50,51,52,53]. In this study, serial deletion of regions flanking *Sp-IAG* 5′ end and SDM analysis of STAT-binding site in pGL3-p*Sp-IAG*3 revealed that STAT-binding site could positively regulate the promoter activity of *Sp-IAG*. An interference of the cultured AG by *Sp-STAT-dsRNA* could effectively decrease the expression of *Sp-IAG* significantly, which further reaffirmed transcriptional activation of STAT in regulating expression of *Sp-IAG*. Recent studies have shown that JAK/STAT signaling is involved in regulating immunity in *S. paramamosain*, and expression of *Sp-STAT* can be significantly affected by various immunostimulants [54]. In this study, it was found that the level of *Sp-STAT* mRNA was significantly decreased in AG treated with rCFSH, suggesting that CFSH can inhibit expression of *Sp-STAT* at gene level, which in turn regulates the expression of *Sp-IAG*.

To our knowledge, this study presents the first attempt to comprehensively investigate the transcriptional mechanism of *IAG* expression in crustaceans. AP-1-binding site, Sox-5-binding site and STAT-binding site can regulate expression of *Sp-IAG*, in which STAT-binding site is involved in transcription regulation of *Sp-CFSH* on *Sp-IAG* expression. So far, CFSH receptor has not been identified in crustacean species, further study on CFSH receptor and its downstream signaling pathways are recommended for the interpretation *Sp-IAG* expression in *S. paramamosain*.

## 4. Materials and Methods

### 4.1. Animal Sources

Mud crabs (*Scylla paramamosain*) were obtained from a local market in Xiamen, Fujian Province, China. In this study, crabs at pre-molt stage in autumn were selected. Crabs (carapace width 11.5–14.3 cm, body weight 250–320 g) were anesthetized and dissected, collecting muscles and AGs at stage III of the gland development [16]. Muscles provided genomic DNA while AGs were used for in vitro incubation study. The crabs were reared in tanks (40 × 40 × 60 cm) with seawater (24 L, salinity 26 ± 1 ppm and temperature 27 ± 2 °C), and fed on the white Pacific shrimp (*Litopenaeus vannamei*) meat. All animal procedures were carried out in strict compliance with the National Institute of Health Guidelines for the Care and Use of Laboratory Animals.

### 4.2. Transcriptional Activity Analysis of Sp-IAG Promoter

The 5′ region flanking *Sp-IAG* was amplified by PCR and then cloned in pMD19-T vector (TaKaRa, Dalian, China). Briefly, this region was digested with KpnI and BamHI (TaKaRa, Dalian, China), and ligated in a vector lacking gene promoters (pEGFP-1, YouBio, Changsha, China). The recombinant vector (pEGFP-p*Sp-IAG*) was transfected on HEK293FT cells to determine transcriptional activity. The HEK293FT cells were obtained from the China Center for Type Culture Collection, Wuhan. After three passages, HEK293FT cells were cultured on 24-well plates in high glucose Dulbecco’s modified eagle medium (DMEM, HyClone, Logan, UT, USA) containing 10% FBS (fetal bovine serum) (Gibco, Grand Island, NY, USA) and 1% Penicillin-Streptomycin (Gibco, USA), at 37 °C for 12 h under 5% CO_2_. Transient transfection of cells was performed using LipofectamineTM 2000 Reagent (Invitrogen, Carlsbad, CA, USA) according to the manufacturer’s protocols. pEGFP-N1 contains cytomegalovirus (CMV) promoter, which effectively turns on the expression of EGFP. pEGFP-1 and pEGFP-N1 were used as negative and positive controls, respectively. After culture for 24 h, the cells were observed under a fluorescent microscope and green fluorescence of the live cells was observed under a Leica fluorescence microscopy imaging system (Leica, Solms, Germany).

### 4.3. Analysis of Promoter Activity by Serial Deletion of 5′ Region Constructs

Sequential deletion of fragments flanking 5′ *Sp-IAG* region were synthesized using primers (Table 1) containing KpnI and XhoI restriction sites (fixing the 3′-terminal and shrinking the 5′-terminal). After digestion with KpnI and XhoI (TaKaRa, Dalian, China), a series of recombinant vectors (p*Sp-IAG*-1 to p*Sp-IAG*-8) were constructed by connecting pGL3-Basic vector (a Luciferase Reporter Vector without promoter, Promega, Madison, WI, USA) with the synthesized fragments described above. The recombinant vectors were verified by sequencing and then transfected on HEK293FT cells using LipofectamineTM 2000 Reagent (Invitrogen, Carlsbad, CA, USA) according to the manufacturer’s protocols [32]. pRL-TK vector was used to normalize the transfection efficiency by co-transfected with the reporter constructs. Cells were collected after a 24 h culture. Firefly and Renilla luciferase activities were measured by Dual-Luciferase Reporter Assay System (Promega, Madison, WI, USA) and chemiluminescence was measured using Varioskan Flash (Thermo Scientific, Waltham, MA, USA). Promoter activity was evaluated by the normalized mean of firefly luciferase action on Renilla luciferase activity.

### 4.4. Analysis of the Promoter Activity of the 5′ Region Flanking Sp-IAG with Site-Directed Mutagenesis (SDM)

SDM was performed by overlap extension PCR with primers containing the mutational bases (the transcription factor binding sites are shown in bold, Table 1), and was used to identify the function of transcription elements. p*spIAG*-2F/*Sp-AP*-R-mut, p*spIAG*-3F/*Sp-STAT*-R-mut, p*spIAG*-6F/*Sp-Sox*-R-mut, and p*spIAG*-8F/*Sp-GATA*-R-mut were utilized in amplifying p*Sp-AP*-5′, p*Sp-STAT*-5′, p*Sp-Sox*-5′, and p*Sp-GATA*-5′; *Sp-AP*-F-mut/*Sp-STAT*-F-mut/*Sp-Sox*-F-mut/*Sp-GATA*-F-mut while p*spIAG*-R was utilized in amplifying p*Sp-AP*-3′, p*Sp-STAT*-3′, p*Sp-Sox*-3′, and p*Sp-GATA*-3′. PCR conditions were 34 cycles, with initial 94 °C for 5 min; and subsequently 94 °C for 30 s, 57 °C for 30 s, 72 °C for 1 min, followed by final extension at 72 °C for 10 min using an ABI 2720 Thermal Cycler (Applied Biosystems). The second reaction was performed in an amplification system containing 2.0 µL 10× Ex Taq Buffer (Mg^2+^ Plus), 0.8 µL dNTP (2.5 mM), 0.1 µL Ex Taq (5U/μL) (TaKaRa, Dalian, China), 2 µL p*Sp-AP*-5′/p*Sp-STAT*-5′/p*Sp-Sox*-5′/p*Sp-GATA*-5, 2 µL p*Sp-AP*-3′/p*Sp-STAT*-3′/p*Sp-Sox*-3′/p*Sp-GATA*-3′, and 12.3 µL water, under initial 94 °C for 5 min; followed by 10 cycles, each at 94 °C for 40 s, 57 °C for 1 min, 72 °C for 1 min, 20 °C for 5 min followed by adding 0.4 µL p*spIAG*-2F/p*spIAG*-3F/p*spIAG*-6F/p*spIAG*-8F and 0.8 µL p*spIAG*-R (10μM) to the amplification system. The second reaction conditions were 35 cycles, each at 94 °C for 40 s, 57 °C for 1 min, 72 °C for 1 min, and 72 °C for 10 min. Amplification products were verified by sequencing. Products with SDM were purified and digested with KpnI and XholI (TaKaRa, Dalian, China), then ligated in pGL3-Basic vector. The reporter vectors were transiently transfected on HEK293FT cells and evaluated for relative luciferase activity by Dual-Luciferase Reporter Assay (Promage, Madison, WI, USA). Reporter vector with normal transcription element and pGL3-basic were used as positive and the negative controls, respectively.

### 4.5. Production of Recombinant CFSH

Recombinant CFSH (rCFSH) was produced as previously described [16]. Briefly, *Sp-CFSH* was amplified and cloned in pET-His (a prokaryotic expression vector). After sequencing and analysis, the recombinant vector was transfected on *E. coli* BL21 (DE3), and cultured in Luria-Bertani medium with 0.8 mM isopropyl-D-thiogalactopyranoside for 8 h at 37 °C. After collection and cleaning, the bacteria were suspended in binding buffer and dissociated by ultrasonic wave. Soluble and insoluble fractions were separated by centrifugation at 12,000× *g* for 10 min at 4 °C. The precipitate containing large amount of rCFSH was resuspended in binding buffer with 8 M urea, then which was analyzed by 15% SDS-PAGE and loaded on a Ni-NTA HisTrapTM FF crude column (GE Healthcare, Danderyd, Sweden) in accordance with the manufacturer’s instructions. Gradient elution was performed with a buffer containing 50 mM imidazole, 100 mM imidazole, 200 mM imidazole and 300 mM imidazole. Eluting solution was collected and examined with 15% SDS-PAGE. Finally, purified rCFSH was denatured with graded urea dialysis and confirmed by Western blotting. The proteins were stored at −80 °C.

### 4.6. Analysis of Gene Expression in AG Treated by rCFSH in Vitro

CFSH has been shown to inhibit expression of *Sp-IAG* in *S. paramamosain*. AGs of one crab were divided into two and cultured according to the established methods [55,56]. First, AGs were rinsed 9 times with saline containing penicillin G (300 IU/mL) and streptomycin (300 µg/mL) (Sigma-Aldrich Co. Louis, MO, USA), then pipetted on a 48-well plate and pre-incubated at 25 °C with 100 μL 2 × L15 medium (Gibco, Grand Island, NY, USA). The medium was changed after one hour for final incubation. Controls were incubated in 2 × L15 medium only while the test samples were incubated in 2 × L15 medium containing 10^−6^ M rCFSH (rCFSH group). AGs were collected after 8 h to determine the effects of rCFSH on *Sp-AP-1/Sp-STAT/Sp-Sox/Sp-IAG* expression. Four biological replicas were prepared in each experiment.

### 4.7. Sp-STAT Knockdown in Vitro

Green fluorescent protein gene (*GFP*), as an exogenous control, was cloned in pSicoR-EGFP vector [56]. As previously described [57], *Sp-STAT* and *GFP* were cloned and purified for synthesis of *Sp-STAT-dsRNA* and *GFP-dsRNA* by T7 and SP6 polymerase (Roche, Mannheim, Germany). AGs of one crab were divided into three. AGs were rinsed 9 times with saline containing penicillin G (300 IU/mL) and streptomycin (300 µg/mL) (Sigma-Aldrich Co. Louis, MO, USA), then pipetted on a 48-well plate and pre-incubated at 25 °C with 100 μL 2 × L15 medium (Gibco, Grand Island, NY, USA). The medium were changed after one hour for final incubation. They were incubated in 2 × L15 medium (positive control), 2 × L15 medium containing 1 µg/mL *GFP-dsRNA* (negative control) or 2 × L15 medium containing 1 µg/mL *Sp-STAT-dsRNA*, respectively. AGs were collected after either 2 or 4 h to evaluate effects of *Sp-STAT* mRNA knockdown. Then, samples with obvious interference efficiency were selected to detect the expression of *Sp-IAG* gene. Four biological replicas were evaluated in the experiments.

### 4.8. Quantification of Gene Expression

Total RNA was extracted from AGs using TRIzol (Invitrogen, USA). Quality and concentration of RNA was measured using a NanoDrop-2000 spectrophotometer (Thermo Fisher Scientific, Waltham, MA, USA), and 200 ng of total RNA was reverse-transcribed with random primer using TransScriptII One-step gDNA Removal and cDNA Synthesis SuperMix (TransGen, Beijing, China) according to the manufacturer’s protocol. The cDNAs were diluted four times for quantitative reverse transcription PCR (qRT-PCR) analysis, with *β-actin* used as a reference gene. Primers in qRT-PCR assay had been previous used as shown in Table 1 [16,54]. Products of the reaction were sequenced to confirm the specificity of qRT-PCR. qRT-PCR was performed with SYBR Select Master Mix (TaKaRa, Dalian, Japan) under the following conditions: an initial 95 °C for 30 s, 40 cycles at 95 °C for 5 s, 58 °C for 30 s, and 72 °C for 30 s. The procedure was carried out in a 7500 Fast Real-time PCR System (Applied Biosystems). Melting curves were analyzed to exclude any non-specific products. qRT-PCR data were calculated by 2^−ΔΔCt^ method.

### 4.9. Statistical Analysis

The 2^−ΔΔCt^ method was used to analyze the qRT-PCR data. The statistical analysis was performed using SPSS 22.0, including Duncan’s multiple range tests and one-way ANOVA, and *p*-value < 0.05 was considered statistically significant.

## Figures and Tables

**Table 1:** The primers used in the present study.

Name	Sequence (5′→ 3′)	Application
*SpIAG*-PF	cggGGTACCTCCAGCTCTTAGGATGCGTCCA	5′-flanking region sequence
*SpIAG*-PR	cgcGGATCCAGGGAGGCCAAAGAAAACGTAG	
p*spIAG*-1F	cggGGTACC CTGAAACTTGTCAACATGCGGAG	serial deletion of 5′-flanking region
p*spIAG*-2F	cggGGTACC CTCTAAGTAAAAATTAGCAAAACAGACG	
p*spIAG*-3F	cggGGTACC GTTCCTGCCTCGTCATATCGC	
p*spIAG*-4F	cggGGTACCACAAATAAAGCGTGTATTCTGTGGTA	
p*spIAG*-5F	cggGGTACC CCGCAGCCAGAACCAATCT	
p*spIAG*-6F	cggGGTACC TGACTTGAACTGTTTCAAGGGAGAG	
p*spIAG*-7F	cggGGTACC TGATTCTAGCGGCGGACTGT	
p*spIAG*-8F	cggGGTACCCACTAGACCTTTTGGTGCTCGC	
p*spIAG*-R	ccgCTCGAGAGGGAGGCCAAAGAAAACGTAG	
*Sp-AP*-F-mut	GTAGAGGTG**TGCAGAC**AGCCCTGACCGAGC	SDM
*Sp-AP*-R-mut	GCTCGGTCAGGGCT**GTCTGCA**CACCTCTAC	
*Sp-Sox*-F-mut	CAGCCGAATACAG**GTCTCGTTAGCCT**AACC	
*Sp-Sox*-R-mut	GGTT**AGGCTAACGAGAC**CTGTATTCGGCTG	
*Sp-GATA*-F-mut	GGGTTGTGTGTCT**GGCTGCATTCTAG**TAAG	
*Sp-GATA*-R-mut	CTTA**CTAGAATGCAGCC**AGACACACAACCC	
*Sp-STAT*-F-mut	CACGCAGT**CGAGATACGAGACGA**CCGCTGA	
*Sp-STAT*-R-mut	TCAGCGG**TCGTCTCGTATCTCG**ACTGCGTG	
*Sp-STAT*F	CACCAGATCAAGGAGTGTGAGCGACA	qPCR
*Sp-STAT*R	GGTGACAAGTGAGGACAGCAAGCGA	
*Sp-IAG*F	ATCCTTTTCCTCCGTTTGCC	
*Sp-IAG*R	TCGGGTCTTCGTCTTGTTCC	
*Sp-cfsh*F	CGTGTCCAGCATTTCTTGCAGTACC	
*Sp-cfsh*R	TCATGTGTCCTATGATGGAGGAACG	
*Sp-ak*F	TTCCTCCACCCTGTCCAACC	
*Sp-ak*R	GAAGCGGTCACCCTCCTTGA	
*Sp-actin*F	CACACTTCACAGACCTTC	
*Sp-actin*R	CACAATGCCATCCTCTAC	
*Sp-STAT-dsF*	CTTGGTGCTCCACACACAACTAAT	*Sp-STAT* knockdown
*Sp-STAT-dsR*	CCATGTGGGGTTATTGGTATCTT	
*Sp-GFP-dsF*	TGGGCGTGGATAGCGGTTTG	*Sp-GFP* knockdown
*Sp-GFP-dsR*	GGTCGGGGTAGCGGCTGAAG	
CFSH-EF	CGCGGATCCTCCTCCATCATAGGACACATGAATTC	rCFSH expression
CFSH-ER	GGACTAGTTTTATTCTCGCTTAAGTCGATGTAG	

## Supplementary Materials

Supplementary materials can be found at https://www.mdpi.com/1422-0067/21/15/5300/s1. Figure S1: Production of recombinant CFSH. A and B: rCFSH after lysis and purification analyzed by 15% SDS-PAGE; C: Western blotting analysis of the rCFSH after purification and renaturation. M: Protein marker. Arrows indicate the target protein product. Supplementary 2: The 5′ flanking region sequence of *Sp-IAG*. The deduced transcriptional start site (G) is marked by red and defined as position 1. The primers used to clone the 5′-flanking region sequence are marked in bold. The restriction sites are marked by underlined. The protective base is represented in lowercase letters.

## Abbreviations

CFSH	crustacean female sex hormone
IAG	insulin-like androgenic hormone
SDM	site-directed mutation
STAT	signal transducers and activators of transcription
AP-1	activator protein 1
RNAi	RNA interfere
EGFP	enhanced green fluorescent protein
rCFSH	recombinant CFSH
AG	androgenic gland
AGH	androgenic gland hormone
IL-17	interleukin-17

## References

[1] Li J., Yu H., Wang W., Fu C., Zhang W., Han F., Wu H. (2019). Genomic and transcriptomic insights into molecular basis of sexually dimorphic nuptial spines in *Leptobrachium leishanense*. Nat. Commun..

[2] Jiang Q., Lu B., Lin D., Huang H., Chen X., Ye H. (2020). Role of crustacean female sex hormone (CFSH) in sex differentiation in early juvenile mud crabs, *Scylla paramamosain*. Gen. Comp. Endocrinol..

[3] Charniaux-Cotton H. (1992). *Arthropoda-Crustacea*: Sexual differentiation. Reprod. Biol. Invert..

[4] Charniaux-Cotton H. (1954). Discovery in, an amphipod crustacean (*Orchestia gammarella*) of an endocrine gland responsible for the differentiation of primary and secondary male sex characteristics. Cr. Acad. Bulg. Sci..

[5] Okuno A., Hasegawa Y., Ohira T., Katakura Y., Nagasawa H. (1999). Characterization and cDNA Cloning of Androgenic Gland Hormone of the Terrestrial Isopod *Armadillidium vulgare*. Biochem. Biophys. Res. Commun..

[6] Khalaila I., Katz T., Abdu U., Yehezkel G., Sagi A. (2001). Effects of Implantation of Hypertrophied Androgenic Glands on Sexual Characters and Physiology of the Reproductive System in the Female Red Claw Crayfish, *Cherax quadricarinatus*. Gen. Comp. Endocrinol..

[7] Sagi A., Khalaila I. (2001). The crustacean androgen: A hormone in an isopod and androgenic activity in decapods. Am. Zool..

[8] Barki A., Karplus I., Khalaila I., Manor R., Sagi A. (2003). Male-like behavioral patterns and physiological alterations induced by androgenic gland implantation in female crayfish. J. Exp. Boil..

[9] Manor R., Aflalo E., Segall C., Weil S., Azulay D., Ventura T., Sagi A. (2004). Androgenic gland implantation promotes growth and inhibits vitellogenesis in *Cherax quadricarinatus* females held in individual compartments. Invertebr. Reprod. Dev..

[10] Rosen O., Manor R., Weil S., Gafni O., Linial A., Aflalo E., Ventura T., Sagi A. (2010). A Sexual Shift Induced by Silencing of a Single Insulin-Like Gene in Crayfish: Ovarian Upregulation and Testicular Degeneration. PLoS ONE.

[11] Ventura T., Rosen O., Sagi A. (2011). From the discovery of the crustacean androgenic gland to the insulin-like hormone in six decades. Gen. Comp. Endocrinol..

[12] Sagi A., Snir E., Khalaila I. (1997). Sexual differentiation in decapod crustaceans: Role of the androgenic gland. Invertebr. Reprod. Dev..

[13] Ventura T., Manor R., Aflalo E., Weil S., Raviv S., Glazer L., Sagi A. (2009). Temporal Silencing of an Androgenic Gland-Specific Insulin-Like Gene Affecting Phenotypical Gender Differences and Spermatogenesis. Endocrinology.

[14] Huang X., Ye H., Huang H., Yang Y., Gong J. (2014). An insulin-like androgenic gland hormone gene in the mud crab, *Scylla paramamosain*, extensively expressed and involved in the processes of growth and female reproduction. Gen. Comp. Endocrinol..

[15] Zhang Y., Qiao K., Wang S., Peng H., Shan Z., Wang K. (2014). Molecular identification of a new androgenic gland-specific insulin-like gene from the mud crab, *Scylla paramamosain*. Aquac..

[16] Liu A., Liu J., Liu F., Huang Y., Wang G., Ye H.-H. (2018). Crustacean Female Sex Hormone from the Mud Crab *Scylla paramamosain* is Highly Expressed in Prepubertal Males and Inhibits the Development of Androgenic Gland. Front. Physiol..

[17] Denny P., Swift S., Brand N., Dabhade N., Barton P., Ashworth A. (1992). A conserved family of genes related to the testis determining gene, SRY. Nucleic Acids Res..

[18] Uwanogho D., Rex M., Cartwright E.J., Pearl G., Healy C., Scotting P.J., Sharpe P.T. (1995). Embryonic expression of the chicken Sox2, Sox3 and Sox11 genes suggests an interactive role in neuronal development. Mech. Dev..

[19] Clarkson M., Harley V.R. (2002). Sex with two SOX on: SRY and SOX9 in testis development. Trends Endocrinol. Metab..

[20] Tevosian S.G., Albrecht K., Crispino J.D., Fujiwara Y., Eicher E.M., Orkin S.H. (2002). Gonadal differentiation, sex determination and normal Sry expression in mice require direct interaction between transcription partners GATA4 and FOG2. Development.

[21] Robert N.M., Tremblay J.J., Viger R.S. (2002). Friend of GATA (FOG)-1 and FOG-2 Differentially Repress the GATA-Dependent Activity of Multiple Gonadal Promoters. Endocrinology.

[22] Tremblay J.J., Viger R.S. (2003). Novel roles for GATA transcription factors in the regulation of steroidogenesis. J. Steroid Biochem. Mol. Boil..

[23] Viger R.S., Taniguchi H., Robert N.M., Tremblay J.J., And N.M.R. (2004). The 25th Volume: Role of the GATA Family of Transcription Factors in Andrology. J. Androl..

[24] Zmora N., Chung J.S. (2014). A Novel Hormone Is Required for the Development of Reproductive Phenotypes in Adult Female Crabs. Endocrinology.

[25] Benderdour M., Tardif G., Pelletier J.-P., Di Battista J.A., Reboul P., Ranger P., Martel-Pelletier J. (2002). Interleukin 17 (IL-17) induces collagenase-3 production in human osteoarthritic chondrocytes via AP-1 dependent activation: Differential activation of AP-1 members by IL-17 and IL-1beta. J. Rheumatol..

[26] Gao C., Liu W., Wang X.R., Liu X.Z., Zhao S.G., Fu S.B. (2009). IL-17 stimulates migration of carotid artery vascular smooth muscle cells in an MMP-9 dependent manner via p38 MAPK and ERK1/2-dependent NF-κB and AP-1 activation. Cell. Mol. Neurobiol..

[27] Wu J., Guo J., Cao Q., Wang Y., Chen J., Wang Z., Yuan Z. (2017). Autophagy impacts on oxaliplatin-induced hepatocarcinoma apoptosis via the IL-17/IL-17R-JAK2/STAT3 signaling pathway. Oncol. Lett..

[28] Du S., Li Z., Xie X., Xu C., Shen X., Wang N., Shen Y. (2020). IL-17 stimulates the expression of CCL2 in cardiac myocytes via Act1/TRAF6/p38MAPK dependent AP-1 activation. Scand. J. Immunol..

[29] Khalaila I., Manor R., Weil S., Granot Y., Keller R., Sagi A. (2002). The eyestalk–androgenic gland–testis endocrine axis in the crayfish *Cherax quadricarinatus*. Gen. Comp. Endocrinol..

[30] Sroyraya M., Chotwiwatthanakun C., Stewart M.J., Soonklang N., Kornthong N., Phoungpetchara I., Hanna P., Sobhon P. (2010). Bilateral eyestalk ablation of the blue swimmer crab, *Portunus pelagicus*, produces hypertrophy of the androgenic gland and an increase of cells producing insulin-like androgenic gland hormone. Tissue Cell.

[31] Zhang X., Huang D., Jia X., Zou Z., Wang Y., Zhang Z. (2018). Functional analysis of the promoter of the molt-inhibiting hormone (mih) gene in mud crab *Scylla paramamosain*. Gen. Comp. Endocrinol..

[32] Liu C.Y., Jia X.W., Zou Z.H., Wang X.W., Wang Y.L., Zhang Z.P. (2018). VIH from the mud crab is specifically expressed in the eyestalk and potentially regulated by transactivator of Sox9/Oct4/Oct1. Gen. Comp. Endocr..

[33] Wallis M.C., Waters P.D., Graves J.M. (2008). Sex determination in mammals—Before and after the evolution of SRY. Cell. Mol. Life Sci..

[34] Ma K., Qiu G., Feng J., Li J. (2012). Transcriptome Analysis of the Oriental River Prawn, *Macrobrachium nipponense* Using 454 Pyrosequencing for Discovery of Genes and Markers. PLoS ONE.

[35] Gao S., Zhang T., Zhou X., Zhao Y., Li Q., Guo Y., Cheng H., Zhou R. (2005). Molecular cloning, expression ofSox5 and its down-regulation ofDmrt1 transcription in Zebrafish. J. Exp. Zoöl. Part B Mol. Dev. Evol..

[36] Schartl M., Schories S., Wakamatsu Y., Nagao Y., Hashimoto H., Bertin C., Mourot B., Schmidt C., Wilhelm D., Centanin L. (2018). Sox5 is involved in germ-cell regulation and sex determination in medaka following co-option of nested transposable elements. BMC Boil..

[37] Li S., Li F., Sun Z., Xiang J. (2012). Two spliced variants of insulin-like androgenic gland hormone gene in the Chinese shrimp, *Fenneropenaeus chinensis*. Gen. Comp. Endocrinol..

[38] Shabgah A.G., Fattahi E., Shahneh F.Z. (2014). Interleukin-17 in human inflammatory diseases. Adv. Dermatol. Allergol..

[39] Kumar P., Monin L., Castillo P., Elsegeiny W., Horne W., Eddens T., Vikram A., Good M., Schoenborn A.A., Bibby K. (2016). Intestinal Interleukin-17 Receptor Signaling Mediates Reciprocal Control of the Gut Microbiota and Autoimmune Inflammation. Immunity.

[40] Subramaniam S., Cooper R.S., Adunyah S.E. (1999). Evidence for the Involvement of JAK/STAT Pathway in the Signaling Mechanism of Interleukin-17. Biochem. Biophys. Res. Commun..

[41] You T., Bi Y., Zhang M., Chen X., Zhang K., Li J. (2017). IL-17 induces reactive astrocytes and up-regulation of vascular endothelial growth factor (VEGF) through JAK/STAT signaling. Sci. Rep..

[42] Jochum W., Passegué E., Wagner E.F. (2001). AP-1 in mouse development and tumorigenesis. Oncogene.

[43] Grötsch B., Brachs S., Lang C., Luther J., Derer A., Schlötzer-Schrehardt U., Bozec A., Fillatreau S., Berberich I., Hobeika E. (2014). The AP-1 transcription factor Fra1 inhibits follicular B cell differentiation into plasma cells. J. Exp. Med..

[44] Li J.K., Nie L., Zhao Y.P., Zhang Y.Q., Wang X., Wang S.S., Liu Y., Zhao H., Cheng L. (2016). IL-17 mediates inflammatory reactions via p38/c-Fos and JNK/c-Jun activation in an AP-1-dependent manner in human nucleus pulposus cells. J. Transl. Med..

[45] He S., Minton A.Z., Ma H.-Y., Stankowska D.L., Sun X., Krishnamoorthy R.R. (2013). Involvement of AP-1 and C/EBPβ in Upregulation of Endothelin B (ETB) Receptor Expression in a Rodent Model of Glaucoma. PLoS ONE.

[46] Baksa K., Parke T., Dobens L.L., Dearolf C.R. (2002). The *Drosophila* STAT Protein, Stat92E, Regulates Follicle Cell Differentiation during Oogenesis. Dev. Boil..

[47] Arbouzova N.I., Zeidler M.P. (2006). JAK/STAT signalling in *Drosophila*: Insights into conserved regulatory and cellular functions. Dev..

[48] Li W.X. (2008). Canonical and non-canonical JAK–STAT signaling. Trends Cell Boil..

[49] Katsuyama T., Comoglio F., Seimiya M., Cabuy E., Paro R. (2015). During *Drosophila* disc regeneration, JAK/STAT coordinates cell proliferation with Dilp8-mediated developmental delay. Proc. Natl. Acad. Sci. USA.

[50] Kiger A.A., Tulina N., Matunis E. (2001). Stem Cell Self-Renewal Specified by JAK-STAT Activation in Response to a Support Cell Cue. Science..

[51] Tulina N. (2001). Control of Stem Cell Self-Renewal in *Drosophila* Spermatogenesis by JAK-STAT Signaling. Science.

[52] Li J., Xia F., Li W.X. (2003). Coactivation of STAT and Ras Is Required for Germ Cell Proliferation and Invasive Migration in Drosophila. Dev. Cell.

[53] Brown S., Zeidler M.P., Hombría J.C.-G. (2006). JAK/STAT signalling in *Drosophila* controls cell motility during germ cell migration. Dev. Dyn..

[54] Deng H., Zhang W., Li J., Li J., Hu L., Yan W., Liu S., He J., Weng S. (2019). A signal transducers and activators of transcription (STAT) gene from *Scylla paramamosain* is involved in resistance against mud crab reovirus. Fish Shellfish. Immunol..

[55] Gong J., Ye H., Xie Y., Yang Y., Huang H., Li S., Zeng C. (2015). Ecdysone receptor in the mud crab *Scylla paramamosain*: A possible role in promoting ovarian development. J. Endocrinol..

[56] Huang X., Ye H., Feng B., Huang H. (2015). Insights into insulin-like peptide system in invertebrates from studies on IGF binding domain-containing proteins in the female mud crab, *Scylla paramamosain*. Mol. Cell. Endocrinol..

[57] Yang Y.N., Shu L., Jiang Q.L., Huang H.Y., Ye H.H. (2018). Does the bone morphogenetic protein 7 inhibit oocyte maturation by autocrine/paracrine in mud crab?. Gen. Comp. Endocr..

