# Supplementary material for: Transcriptional Inhibition of Sp-IAG by Crustacean Female Sex Hormone in the Mud Crab, Scylla paramamosain
Source: Int J Mol Sci. 2020 Jul 26;21(15):5300. doi: 10.3390/ijms21155300 (PMC7432471; doi:10.3390/ijms21155300)
Supplement: Supplementary file 1 [file ijms-21-05300-s001.pdf]

Supplementary 1. Production of recombinant CFSH

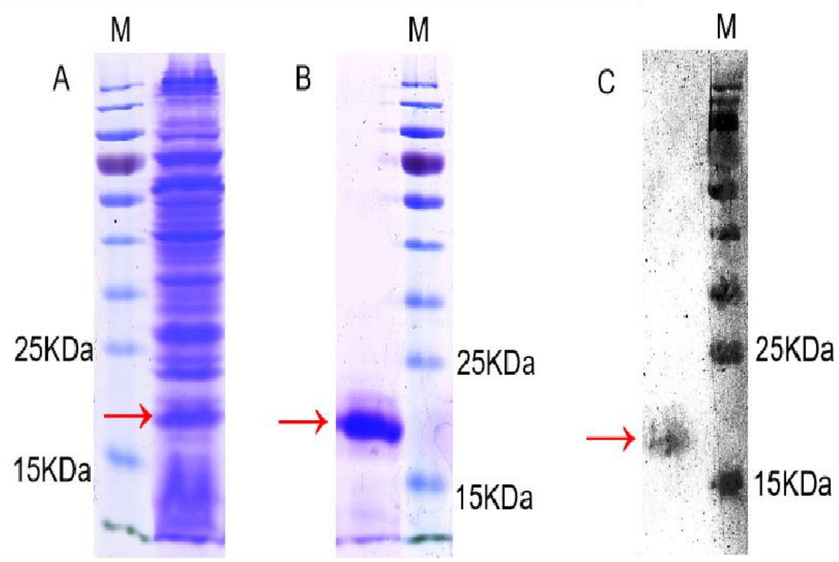

Figure S1. Production of recombinant CFSH. A and B: rCFSH **after lysis and** purification analyzed by 15 % SDS-PAGE; C: Western blotting analysis of the rCFSH after purification and renaturation. M: Protein marker. Arrows indicate the target protein product.

Supplementary 2. The 5' flanking region sequence of *Sp-IAG*.

> 5′flanking region sequence of *Sp-IAG*

cggGGTACC**TCCAGCTCTTAGGATGCGTCCA**CACGGTCGAACACTGTCTGACAAACACTGTTAC
CGTATCATAAGACGAAGCAGGATGACGTCATACTACGGACACGGGCAGTGTCCACTAGACCTTT
TGGTGCTCGCTGGTGACGTCATTGACGCTCCTTTCCGCTTCCCATTGTTAACTGGTAACAGCGGGT
GTTCGACGGACAATGTTCGACAGTGTGGACACGCCCTTGACTATCTTGCTCAAACATTTGCCACC
ACCAGTTGTCTCTGTGCACACGACAAGCCCTCCACATCATCCTGTTTTTAACCTGTGCCGTCTTGA
TATTACAGGCCATTCGTACGAGTAAGTTCGTATTCTCCAACATTTCGGCGTCTTATTTCGGTTACTT
TTAACAATCAAGATATTAGGGTATTTGCGGACGCTTTCATGATTCTAGCGGCGGACTGTCTTCTGC
CGATCTCTCTCTTCGCCGCAGGGTTGTGTGTCTGGCTATCTTCTAGTAAGATTTCTTTCACGCTAAT
TGTTCTTCTGGTCTTACTAACTTCATGCCTCCTTCACTCCCGCTTCCTCACTACACAAGGCTTTCTA
CATTTTCTGATCCCTATTTTGTTCACCTCCATAATGCAAGAGTTTTTTTCATTTCTTCTGCTGAGAA
ACTCTGGAATTCTCTGTTTCTGTATTTCTTTTTACCTTTGACTTGAACTGTTTCAAGGGAGAGGTTG
CAACACATTTCTCTAGTTTTGAGTAATCTTTCGTTGATCTGTACTCTACTTGGACTGGCGCCTTCGG
TAGCCCCTCCCCCTCCCTTTTTTTTTAATATAACTTTTTTGTTGGCCGCAGCCAGAACCAATCTTAC
GTAAAAAAGTTTAACCAAGATTCTGTACCATCAATGGAAGAAGCACTCATGATACTCACAAGTC
AATCACCTCTGTTGTCTCTGAAGGTAGTCTGTCTAAGAGTCTGAAGTGATGAACACGGGCCGTAC
AGACAAGGTCAATGGTCACAGCAACAAAGAGTAACAGTTACACTTACTTTCACTGTATTCAGCG
TTCTCCCTCAGTCTTCAATAACCTGAAATGCTAAAAATCTATCAGTAATATATTCTATAAAGAGATT
ATCACTCAGCCGAATACAGGTATTGTTAACCTAACCTAGCTTAACCTAATGTATCTAACCTATTTT
GTAAGAACATTGTCATTCAGCAGATTAGTCTAAAATTAAGGGAAGAAGACGAACACAGTGGAA
GGGAGTGTAGGCTAATCGACTTCGCTCAGTGTACACAGGCGTATGACCCGAAACATACGGAATG
GTTGCCAACTTGTTATATTTTCAGTTAGTTTGAATAGAAAACATGGCGTTGTTTTGTAAATACTGTA
TAGGGAGTGTGTGTGTGTGTGTGTGTGTAAGGCGTCATAGAAAAGCTCATTGAAGTGTACAAAC

AAAAACAAATAAAGCGTGTATTCTGTGGTAAATAAAAGTTTTCGTTTCGTTTTGAAATAATATCTT
TCCCACTCAGCAATGCGACCATATTGCCAACACACACACACACACACACACACACACACACATCAT
TTATCACAACAATCAAGCGCTACAAGTCACTAATTAACTGTTACACCCTCGCTGGCTTTATCAAC
CACATTGCCGATAGTGTTTCCTGACCACCGAGCACTGGACAGGAATGCACCGTTCTTCCCCTTGA
CACCCTCTCATTGTTCCTGCCTCGTCATATCGCTCCCCTCTTCTCCAACGACCTCGCTTCTCGTATT
TCTGTCGTACGGCGCCATATGACCTTGCTGATGCGGCGCAACTCACCTCAGTCAAACAAAGGTA
ATTTTCTTGCACTCTAAGTAAAAATTAGCAAAACAGACGAAAATATCAAAACGCCAAACACACG
TAAAAGATAAAAAGATGTAGATCGAATTGTTACTAAAATAATTCTTTCTCACTATCGTTCTTTATA
GTACGAAGAAAGAGAAAGGAAAGGAGGGAAAGAAAGGAAAGGGAAGGGTAGAGAAACATG
ACAGGAAAGAAGAAAAAAGGAGGTAATGAAAGGGAAGAAAGAAGAAAAGGAAAGAGAAGA
AAAGGAAGGGAAGGGAGGGGAAGAGAAAAGCGACGTACAAGAGGTAAAGGGGAAGAGTTTT
TTTTTTCTATTACGTGAAATCCTTCTTAAACTATTACCAGAATCATGAAACCACCATTGAAAACCT
GAAATAATTTCCAATAGAACCTGTTCACGCAGTCGAGATACGAGGAAACCGCTGAAACTTGTCA
ACATGCGGAGATTAAGGCTGAGACAAAACACAAAATAGAAAAGTAGAGGTGTTAATCAAGCCC
TGACCGAGCACACTGCTGTCACTGAGGGGAGGGCGAAGAGCAACAATCAGCACCTTAAAACAA
TTGCTGCTTGAACGGAGACGTCACAATGTCAAAACAAGTCAAGGTTCTGAGCATACAATGGCCT
CTTGGTGGTTATGGTGCCAACACTCTGGGTATATAAGCTTACACATGGAGCTCCTTGGCCTGCGGT
GCATCATCAGGAGCTGCTCTTGGCACTTGACACCTCGGCACGGCACGGCACGGCACACCTTCCG
CCCGCCACGCCCTTCCGCCACCTCTTCCTTTTCTAGTCAGCAGCAACTACTTCTCCTGGCCTGTA**C**
TACGTTTTCTTTGGCCTCCCTGGATCCgcg

Supplementary 2. The 5' flanking region sequence of *Sp-IAG*. The deduced transcriptional start site (G) is marked by red and defined as position 1. The primers used to clone the 5′-flanking region sequence are marked in bold. The restriction sites are marked by underlined. The protective base is represented in lowercase letters.
